# An internet-based, multicenter, large-sample survey: knowledge, attitudes, and practices and pathway mechanisms among caregivers of children with central precocious puberty

**DOI:** 10.1530/EC-26-0351

**Published:** 2026-10-06

**Authors:** Shuxia Ding, Wenli Lu, Xiao Yu, Jing Chen, Shuangxi Cheng, Wei Gu, Qiao Wang, Gaohui Zhu, Pingping Zhang, Xiaoyu Ma, Caihong Liu, Yanyan Lin, Zhuannan Jiang, Yeqing Yuan, Jinglu Jin, Yu Pan, Yan Sun, Xiaoping Luo

**Affiliations:** ^1^ Department of Pediatric Endocrinology, Genetics and Metabolism, Women and Children’s Hospital of Ningbo University, Ningbo, China; ^2^ Department of Pediatrics, Ruijin Hospital, Shanghai Jiao Tong University School of Medicine, Shanghai, China; ^3^ Department of Pediatrics, Tongji Hospital, Tongji Medical College, Huazhong University of Science and Technology, Wuhan, China; ^4^ Hubei Provincial Key Laboratory of Pediatric Genetic Metabolic and Endocrine Rare Diseases, Wuhan, China; ^5^ Hubei Provincial Clinical Research Center for Children’s Growth and Development and Metabolic Diseases, Wuhan, China; ^6^ State Key Laboratory for Diagnosis and Treatment of Severe Zoonotic Infectious Disease, Wuhan, China; ^7^ Department of Child Health, Department of Pediatrics, Women and Children’s Hospital, School of Medicine & School of Public Health, Xiamen University, Xiamen, China; ^8^ Department of Child Health, Dongguan Maternal and Child Health Care Hospital, Dongguan, Guangdong Province, China; ^9^ Department of Endocrinology, Genetics and Metabolism, Children’s Hospital of Nanjing Medical University, Nanjing, China; ^10^ Department of Endocrinology, Genetics and Metabolism, Beijing Children’s Hospital, Capital Medical University, Beijing, China; ^11^ Department of Endocrinology, Children’s Hospital of Chongqing Medical University, Chongqing, China; ^12^ Chongqing Key Laboratory of Child Rare Diseases in Infection and Immunity, Chongqing, China; ^13^ National Clinical Research Center for Children and Adolescents’ Health and Diseases, Chongqing, China; ^14^ Ministry of Education Key Laboratory of Child Development and Disorders, Chongqing, China; ^15^ Department of Pediatrics, Shandong Provincial Hospital Affiliated to Shandong First Medical University, Jinan, Shandong Province, China

**Keywords:** central precocious puberty, caregivers, knowledge-attitude-practice, internet-based survey, patient-reported outcome, path analysis

## Abstract

Central precocious puberty (CPP) requires long-term treatment and follow-up, during which caregivers play a central role in treatment adherence and family decision-making. This multicenter, cross-sectional, internet-based survey aimed to characterize knowledge, attitudes, and practices (KAP) among caregivers of children with CPP and to examine determinants and pathway relationships among these domains. Caregivers of children aged 2–16 years with CPP were recruited from eight pediatric endocrinology centers in China. An anonymous online questionnaire based on the disease-specific PRO-CaregiverCPP scale was administered. Knowledge scores, positive attitudes, and good practices were assessed using predefined criteria. Internal consistency was evaluated using Cronbach’s alpha. Multivariable linear and logistic regression models were used to identify factors associated with knowledge scores, positive attitudes, and good practices. Observed-variable path analysis was performed to estimate direct and indirect associations among KAP domains. A total of 1,471 caregivers were included. The mean knowledge score was 6.31 ± 2.25; 72.5 and 58.8% of caregivers demonstrated positive attitudes and good practices, respectively. Higher knowledge scores were independently associated with greater odds of positive attitudes and good practices. Among caregivers of treated children, 3-monthly injections were associated with higher knowledge scores and higher proportions of positive attitudes and good practices than monthly injections. Path analysis showed that knowledge was directly associated with practices and indirectly associated with practices through positive attitudes. These findings indicate a structured KAP pattern in CPP caregiving and support physician-led education and follow-up strategies to improve caregiver engagement and long-term disease management.

## Introduction

Central precocious puberty (CPP) is defined as the premature onset of pubertal development resulting from the early activation of the hypothalamic–pituitary–gonadal axis (HPGA). It typically manifests as the progression of secondary sexual characteristics before the age of 8 years in girls and 9 years in boys, accompanied by clinical challenges such as advanced bone age and compromised adult height potential (1, 2, 3, 4). In recent years, population-based studies and health service data from multiple countries have indicated an upward trend in the reporting and diagnosis of precocious puberty or early pubertal development. Systematic reviews and meta-analyses suggest that the global prevalence and disease burden of this condition are significant (5). In China, population studies have reported relatively high prevalence rates; furthermore, multiple cohort studies and meta-analyses suggest that early-onset puberty or CPP is not uncommon and is associated with factors such as weight status (6, 7, 8). Similar trends have been observed internationally; for instance, large-scale data from South Korea reported a continuous increase in clinic visits and diagnoses for precocious puberty in recent years (9, 10). Collectively, this evidence suggests that CPP has become a critical health issue in pediatric endocrine clinics, requiring long-term management and exerting a sustained impact on families.

The standard treatment for CPP centers on gonadotropin-releasing hormone analogs (GnRHa), which aim to suppress the premature activation of the HPGA, slow bone age progression, and improve lifelong health trajectories (11, 12, 13). However, CPP management is characterized by its long-term nature and context dependency: children require regular injections, follow-up visits, and growth monitoring, while caregivers must continuously navigate complex trade-offs between efficacy and safety, convenience and economic burden, and the time costs associated with academic schedules and medical visits. Concurrently, early pubertal development may lead to psychosocial cascading effects. Research indicates an association between CPP or early puberty and mental health risks in children and adolescents, involving outcomes such as anxiety, depression, and behavioral problems. Systematic reviews of population-based studies also emphasize the complex and inconsistent relationship between pubertal hormonal changes and mental health, highlighting the need to understand biological shifts within broader familial and social contexts (12, 14, 15).

In the real-world management of CPP, caregivers serve not only as the executors of treatment but also as the primary agents for information acquisition, risk comprehension, and shared decision-making (SDM). Limited evidence suggests that ‘limited health literacy’ is not uncommon among parents of children with CPP, and deficiencies in communication and interaction skills are associated with higher medical decisional conflict (16). Furthermore, research on medication adherence in CPP indicates that factors influencing compliance often cluster at the caregiver level – such as understanding of treatment, concerns, and management capacity – that in turn affect follow-up and disease control. These findings imply that emphasizing ‘standardized treatment’ from a purely medical perspective is insufficient to explain or improve variations in long-term management. Instead, there is an urgent need for a caregiver-centered assessment tool facilitated by physician leadership to identify key gaps in knowledge, beliefs, and behavioral execution. This instrument will provide a basis for precision health education and long-term physician-led management.

The knowledge, attitudes, and practices (KAP) framework is widely used in health education and behavioral intervention research to characterize the associative pathways between knowledge levels, attitudes or beliefs, and behavioral practices. However, the quality of its application in disease-specific contexts depends heavily on the scientific rigor and reproducibility of the measurement tools (17). For CPP, previous research has largely focused on epidemiological trends or clinical outcomes, lacking a systematic measurement of the ‘cognition–attitude–behavioral execution’ chain during the caregiving process. Moreover, existing scales are mostly generic tools or nonstandardized, self-designed questionnaires that fail to capture the highly contextualized caregiving experiences and decision-making trade-offs inherent in long-term CPP management. Consequently, developing and validating a disease-specific tool for caregivers of children with CPP and systematically evaluating KAP status and its determinants in a large sample carry clear clinical and public health significance. Regarding psychometrics, the Consensus-based Standards for the Selection of Health Measurement Instruments (COSMIN) has proposed standards for developing and evaluating patient-reported outcome (PRO) tools centered on content validity, providing an operational pathway for constructing precise, physician-calibrated scales (18, 19).

Furthermore, internet-based questionnaires offer a feasible route for obtaining large-sample, cross-regional caregiver data, particularly for studies requiring coverage of multicenter, multi-province populations. Meanwhile, online surveys face methodological challenges such as selection bias, duplicate or fraudulent data, and inconsistent response quality. Therefore, it is essential to adhere to established reporting standards for web-based surveys and quality control strategies to enhance data credibility and research reproducibility (20). In this digital landscape, physicians play an important role in maintaining clinical rigor and guiding the ethical implementation of emerging AI-driven follow-up tools.

Against this background, this study represents a pioneering multicenter initiative to develop and validate a specific experience scale for caregivers of children with CPP (PRO-CaregiverCPP). Using the core KAP modules of this scale, the study systematically describes the overall characteristics of caregiver knowledge, attitudes, and practices. Furthermore, it identifies key determinants influencing KAP and explores the associative mechanisms between these components within a theoretical framework. By combining disease-specific psychometric tools with a large-scale online survey, this study aims to provide a transformative and translatable evidence base for physician-led family education, adherence support, and SDM interventions in the long-term management of CPP.

## Methods

### Study design

This study employed a multicenter, cross-sectional survey design to evaluate the levels of KAP among caregivers of children with CPP, identify key determinants, and examine potential mechanistic associations among these components. The research was conducted across pediatric endocrine specialist clinics and follow-up settings in eight tertiary hospitals in China. Researchers at each center followed a standardized protocol for participant screening and data collection. An electronic, anonymous, self-administered questionnaire was used for data collection. The system incorporated mandatory items and logic validation to minimize missing data and invalid entries, with predefined rules established to identify and exclude duplicate submissions.

### Participants and sampling

The study population consisted of primary caregivers of children with CPP, defined as family members (mother, father, grandparents, or other guardians) bearing core responsibility for medical decision-making and daily management. Inclusion criteria were caregivers aged 18 years or older who were capable of reading, understanding, and independently completing the questionnaire; a clinical diagnosis of CPP in the child; a child’s age between 2 and 16 years (inclusive); and the caregiver’s provision of electronic informed consent. Exclusion criteria were missing data in core KAP items and palpably substandard responses (e.g. logical contradictions in key variables or system-detected duplicate submissions). A complete-case analysis strategy was adopted, whereby only samples with complete core KAP items were included in regression and path analyses.

### Instrument development and content validity

This study utilized the ‘Patient-Reported Outcome Scale for Caregivers of Children with Central Precocious Puberty’ (PRO-CaregiverCPP), a disease-specific instrument developed by the research team. The development process strictly adhered to the COSMIN framework, prioritizing content validity. Items were generated, screened, and refined through a systematic literature review, semi-structured interviews with caregivers, multicenter clinical expert panel discussions, two rounds of Delphi consultations, and cognitive interviews using the think-aloud method. This ensured that items covered key domains of the CPP caregiving experience, utilized clear language, and maintained contextual relevance. The final scale comprises 38 core items, structured into a core KAP module and an extended assessment module. The complete list of items and response formats is provided in Supplementary Table S1 (see section on Supplementary materials given at the end of the article). The core KAP module is designed to quantify caregivers’ fundamental disease literacy and behavioral execution. The extended assessment module provides a more comprehensive characterization of family burden and treatment preferences; it includes assessments of quality-of-life impacts (adapted from the PedsQL™), economic burden, and decision-making weights for treatment regimens (such as safety and convenience), thereby facilitating an expanded analysis and interpretation of the overall caregiving experience (21, 22).

### Psychometric validation overview of the scale

To ensure the structural stability and measurement reliability of the PRO-CaregiverCPP, structural validity, reliability, and measurement invariance were evaluated using a multicenter validation sample. The sample was randomly split into two equal halves for exploratory and confirmatory structural testing. Exploratory factor analysis (EFA), combined with parallel analysis, supported a stable multidimensional structure, with all items achieving loadings on theoretical factors that met established thresholds. Confirmatory factor analysis (CFA) demonstrated a good model fit: *χ*^2^/df = 2.78, RMSEA = 0.049, CFI = 0.951, TLI = 0.947. Internal consistency and test–retest stability reached high levels (total scale Cronbach’s alpha = 0.912; dimension-specific alpha range = 0.796–0.878; McDonald’ s omega = 0.772–0.883; and test–retest ICC = 0.891). At the item level, a graded response model (GRM) based on item response theory (IRT) was employed to evaluate item parameters, with discrimination indices falling within the ideal range (*a* = 1.51–2.79). Differential item functioning (DIF) testing demonstrated robust measurement invariance of the scale across gender groups (Δ*R*^2^ < 0.02). The aforementioned evidence supports the use of the core KAP module for inferential analysis in this real-world sample.

### Variable definitions and scoring rules

The knowledge (K) dimension consists of eight binary items (Q1–Q8); responses of ‘clear/understood’ are scored as 1, while ‘unclear/not understood’ are scored as 0. The total score ranges from 0 to 8, with higher scores indicating a more robust foundation of disease cognition. The attitude (A) dimension comprises four items rated on a 5-point Likert scale (Q9–Q12), with scores ranging from 1 (Strongly disagree) to 5 (Strongly agree). These items are categorized based on their theoretical constructs into positive attitudes (Q9 and Q11) and perceived barriers/concerns (Q10 and Q12). To facilitate binary outcome modeling, positive attitude was further operationalized as a binary variable: a sub-dimension total score of ≥7 was defined as a positive attitude (A_positive = 1), while a score of <7 was defined as a non-positive attitude (A_positive = 0). The practice (P) dimension centers on the execution of caregiving behaviors and includes four frequency-based 5-point Likert items (Q13–Q16), scored from 1 (Never) to 5 (Always). The total score ranges from 4 to 20, with higher scores representing more thorough behavioral execution. Given that Q17 reflects injection-related challenges and perceived difficulties – a construct closer to barrier experience than behavioral execution – this item was excluded from the total practice score to maintain construct purity and scoring consistency. The binary outcome for practice was defined as good practice (P_good): a total practice score of ≥15 was classified as good practice (P_good = 1), and a score of <15 as non-good practice (P_good = 0). Sociodemographic and clinical variables for both caregivers and children were collected as covariates. These included the caregiver’s relationship to the child, caregiver gender, age group, educational attainment, monthly household income, place of residence (province), child’s gender, ethnicity, child’s age, disease duration, current treatment status, treatment duration, and frequency of administration. Variable grouping was conducted according to the predefined categories in the questionnaire.

### Statistical analysis

Statistical analysis primarily comprised descriptive analysis, reliability assessment, multivariable regression modeling, and path effect decomposition. First, descriptive statistics were performed for the baseline characteristics of caregivers and children; continuous variables were expressed as mean ± standard deviation or median (interquartile range), while categorical variables were presented as frequencies and percentages. Subsequently, the distribution of KAP items was described: the correct response rate was reported for each knowledge item, and the proportion of each response level was reported for attitude and practice items. Overall levels for the total knowledge score, attitude sub-dimension scores, and total practice score were calculated. Internal consistency reliability was assessed using Cronbach’ s alpha, combined with item–total correlations for scale consistency diagnosis. For inferential analysis, a multivariable linear regression model was constructed with the knowledge score (K_score) as a continuous outcome, reporting regression coefficients (β) and their 95% confidence intervals (95% CI). Multivariable logistic regression models were developed for binary outcomes, namely, positive attitude (A_positive) and good practice (P_good), reporting odds ratios (OR) and their 95% CI. To control for the potential influence of knowledge on attitudes and behaviors, K_score was included in the set of covariates for both attitude and practice models. Variable selection was performed using a stepwise method based on the Akaike Information Criterion (AIC) to obtain final models, and multicollinearity was assessed using the variance inflation factor (VIF). To examine the mechanistic associations among knowledge, attitudes, and practices, an observed-variable path analysis model was constructed. In this model, knowledge score served as the independent variable, positive attitude as the mediator, and practice as the outcome variable to estimate direct, indirect, and total effects. Confidence intervals for indirect effects were obtained using the nonparametric bootstrapping method. All analyses were performed using R version 4.4.1 and Python version 3.11. All tests were two-sided, and a *P* < 0.05 was considered statistically significant.

## Results

### Participant flow and baseline characteristics

A total of 1,520 questionnaires were recovered in this study. Following screening based on the predefined age range, 1,476 caregivers of children aged 2–16 years were initially included. After further excluding five cases with incomplete core KAP items, the final analytical sample comprised 1,471 participants (Fig. 1). The baseline characteristics of the study population are presented in Table 1. Caregivers were predominantly mothers (62.2%), and 67.9% of all caregivers were female. The age of caregivers was primarily concentrated in the 30–39 years (58.5%) and 40–49 years (33.1%) brackets. Regarding educational attainment, the majority held a bachelor’s degree (50.7%). Monthly household income was most frequently reported in the ranges of 5,000–10,000 CNY (34.1%) and 10,000–20,000 CNY (30.4%). The sample distribution covered multiple provinces, with relatively higher proportions from Guangdong, Zhejiang, and Jiangsu. Among the children, females predominated (72.4%), and the majority were of Han ethnicity (98.2%). The mean age of the children was 9.48 ± 2.21 years, with a mean disease duration of 23.37 ± 19.64 months. Most children were currently receiving treatment (80.6%). Among those undergoing treatment, the duration was primarily <1 year (55.2%) or 1–2 years (34.9%), and the most common administration frequency was ‘one injection per month’ (60.2%).

**Figure 1:**
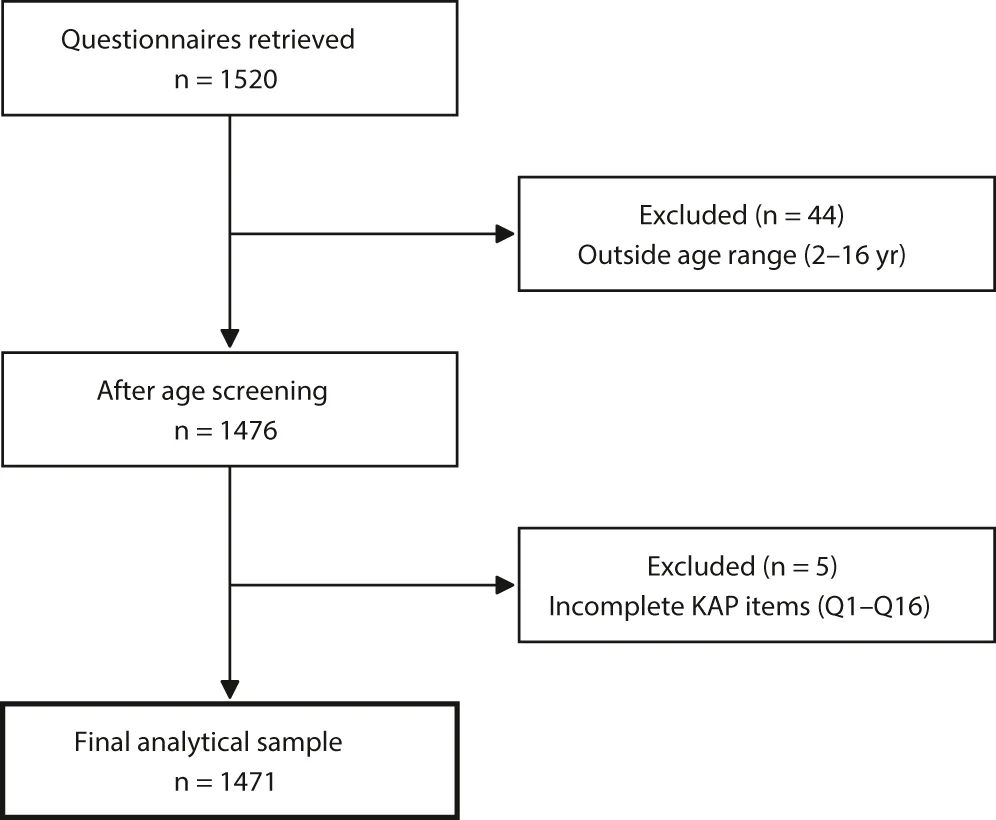
Study flow diagram for the analysis sample.

**Table 1:** Participant and child characteristics (analysis sample, *n* = 1,471). Categorical variables are presented as *n* (%); continuous variables are presented as mean ± SD.

Variable/category	Values
Caregiver characteristics	
Relationship to child	
Mother	915 (62.2)
Father	439 (29.8)
Grandparents	56 (3.8)
Other	61 (4.1)
Gender	
Female	999 (67.9)
Male	472 (32.1)
Age group, years	
<30	51 (3.5)
30–39	861 (58.5)
40–49	487 (33.1)
≥50	72 (4.9)
Educational attainment	
Junior high school or below	104 (7.1)
Senior high or vocational school	175 (11.9)
Junior college	306 (20.8)
Bachelor’s degree	746 (50.7)
Master’s degree or above	140 (9.5)
Monthly household income (CNY)	
<5,000	202 (13.7)
5,000–10,000	501 (34.1)
10,000–20,000	447 (30.4)
20,000–30,000	190 (12.9)
>30,000	131 (8.9)
Region (top 10 provinces)	
Guangdong	237 (16.1)
Zhejiang	220 (15.0)
Jiangsu	197 (13.4)
Fujian	106 (7.2)
Hubei	102 (6.9)
Henan	86 (5.8)
Shandong	81 (5.5)
Liaoning	81 (5.5)
Shanghai	80 (5.4)
Beijing	72 (4.9)
Other provinces	409 (27.8)
Child characteristics	
Gender	
Female	1,065 (72.4)
Male	406 (27.6)
Ethnicity	
Han	1,445 (98.2)
Minority	26 (1.8)
Age, years	9.48 ± 2.21
Disease duration, months	23.37 ± 19.64
Currently receiving treatment	
Yes	1,185 (80.6)
No	286 (19.4)
Treatment duration*	
<1 year	654 (55.2)
1–2 years	414 (34.9)
2–3 years	99 (8.4)
>3 years	18 (1.5)
Administration frequency*	
Monthly injection	710 (59.9)
Three-month depot injection	468 (39.5)
Other	7 (0.6)

Unless otherwise indicated by *, percentages are calculated with *n* = 1,471 as the denominator. *Percentages are calculated within the subsample currently receiving treatment (*n* = 1,185). Region refers to the provincial distribution; only the top 10 provinces are listed individually, with the remainder aggregated as other provinces.

CNY, Chinese yuan.

### KAP item distribution and internal consistency reliability

The summary of overall KAP levels and internal consistency reliability is presented in Table 2. The mean knowledge score (range: 0–8) was 6.31 ± 2.25, demonstrating good internal consistency (Cronbach’s alpha = 0.851). The correct response rates for individual knowledge items ranged from 69.4 to 88.8%, indicating variations in the mastery of different knowledge points (Supplementary Fig. S1). Within the attitude dimension, the mean positive attitude score (range: 2–10) was 7.41 ± 1.67 (alpha = 0.762). Based on the predefined threshold (≥7), the proportion of caregivers demonstrating a positive attitude was 72.5%. The mean score for barriers/concerns (range: 2–10) was 6.89 ± 1.71 (alpha = 0.653), suggesting that the internal consistency of this sub-dimension reached an acceptable level (Table 2). The distribution of responses for attitude items is illustrated in Supplementary Fig. S2. The mean practice score (range: 4–20) was 14.76 ± 2.92 (alpha = 0.792), with 58.8% of participants classified as having ‘good practice’ according to the threshold (≥15). The frequency distribution of practice items is shown in Supplementary Fig. S3. Descriptive comparisons of K, A, and P across different demographic subgroups are provided in Supplementary Table S2. Notably, among caregivers of children receiving GnRHa treatment with comparable dosage forms, those in the three-month formulation group demonstrated higher knowledge scores (6.97 ± 1.59 vs 6.60 ± 1.93), a greater proportion of positive attitudes (82.1 vs 76.3%), and a higher prevalence of good practice (68.4 vs 61.0%) than those in the one-month formulation group.

**Table 2:** Overall KAP level and internal consistency reliability (*n* = 1,471).

Dimension/measure	Score (mean ± SD, range)	Cronbach’s α	Dichotomized proportion
Knowledge (K_score, 0–8)	6.31 ± 2.25 (0–8)	0.851	—
Attitude – positive (A_pos_score, 2–10)	7.41 ± 1.67 (2–10)	0.762	72.5% (cut-off ≥ 7)
Attitude – barriers/concerns (A_bar_score, 2–10)	6.89 ± 1.71 (2–10)	0.653	—
Practice (P_score, 4–20)	14.76 ± 2.92 (4–20)	0.792	58.8% (cut-off ≥ 15)

K_score was calculated as the sum of binary-scored items Q1–Q8. A_pos_score and A_bar_score were derived from summed Likert-scale items. P_score was computed by summing the frequency-based Likert responses for items Q13–Q16. Predefined dichotomization thresholds were applied to construct outcome variables for regression analyses.

### Correlation among continuous KAP scores

The correlations between the continuous scores of the KAP dimensions are summarized in Supplementary Table S4 and Supplementary Fig. S4. The knowledge score showed a moderate positive correlation with the practice score (*r* = 0.390) and a weak positive correlation with the positive attitude score (*r* = 0.186). Furthermore, a moderate positive correlation was observed between the positive attitude score and the practice score (*r* = 0.408). In contrast, the barriers/concerns sub-dimension score demonstrated a moderate correlation with the positive attitude score (*r* = 0.455) and a weak correlation with the practice score (*r* = 0.169). Its correlation with the knowledge score was negligible (*r* = −0.024).

### Multivariable regression analysis: determinants of knowledge, positive attitudes, and good practices

The multivariable models for the three outcome domains are summarized in Table 3, with key effect estimates illustrated in the forest plot (Fig. 2). The final model specifications derived from stepwise selection are presented in Supplementary Table S3. In the multivariable linear regression model with knowledge score as the outcome, treatment regimen was independently associated with knowledge level: compared with caregivers receiving monthly injections, those receiving injections every three months had significantly higher knowledge scores (*β* = 0.416, 95% CI: 0.208–0.623; *P* < 0.001). Male caregivers, compared with female caregivers, exhibited lower knowledge scores (*β* = −0.427, 95% CI: −0.645 to −0.209; *P* < 0.001). Relative to caregivers with a bachelor’ s degree, lower educational attainment – including junior high school or below (*β* = −0.721), senior high or vocational school (*β* = −0.455), and junior college (*β* = −0.347) – was consistently associated with lower knowledge scores (all *P* ≤ 0.011). Caregivers aged 40–49 years demonstrated higher knowledge scores compared with those aged 30–39 years (*β* = 0.273; *P* = 0.016), whereas no statistically significant differences were observed for caregivers aged < 30 years or ≥50 years.

**Table 3:** Multivariable regression models for knowledge, positive attitude, and good practice (treated caregivers, *n* = 1,185).

Predictor domain/predictor (reference)	Model K (OLS)		Model A (logistic)		Model P (logistic)	
	β (95% CI)	*P*	OR (95% CI)	*P*	OR (95% CI)	*P*
Treatment regimen						
Every 3 months vs monthly	0.416 (0.208–0.623)	<0.001	1.370 (1.015–1.849)	0.040	1.274 (0.978–1.659)	0.073
Knowledge						
K_score, per 1-point increase	—	—	1.198 (1.115–1.287)	<0.001	1.483 (1.372–1.603)	<0.001
Caregiver gender						
Male vs female	−0.427 (−0.645 to −0.209)	<0.001	—	—	0.660 (0.503–0.866)	0.003
Caregiver age						
<30 vs 30–39	−0.143 (−0.836 to 0.551)	0.687	—	—	—	—
40–49 vs 30–39	0.273 (0.052–0.494)	0.016	—	—	—	—
≥50 vs 30–39	−0.393 (−0.910 to 0.123)	0.135	—	—	—	—
Caregiver education						
Junior high or below vs bachelor	−0.721 (−1.161 to −0.281)	0.001	0.921 (0.533–1.593)	0.769	—	—
Senior high/vocational vs bachelor	−0.455 (−0.791 to −0.119)	0.008	1.721 (1.030–2.875)	0.038	—	—
Junior college vs bachelor	−0.347 (−0.615 to −0.078)	0.011	0.735 (0.517–1.043)	0.085	—	—
Master’s or above vs bachelor	−0.015 (−0.392 to 0.362)	0.938	0.947 (0.569–1.575)	0.833	—	—
Monthly household income (CNY)						
<5,000 vs 10,000–20,000	−0.303 (−0.660 to 0.053)	0.095	—	—	1.124 (0.741–1.703)	0.582
5,000–10,000 vs 10,000–20,000	0.184 (−0.078 to 0.445)	0.168	—	—	1.264 (0.918–1.740)	0.152
20,000–30,000 vs 10,000–20,000	0.039 (−0.288 to 0.365)	0.816	—	—	1.465 (0.963–2.229)	0.074
>30,000 vs 10,000–20,000	0.284 (−0.089 to 0.658)	0.136	—	—	1.931 (1.176–3.170)	0.009
Child age						
Per 1-year increase	0.081 (0.029–0.133)	0.002	—	—	—	—
Ethnicity						
Minority vs Han	—	—	—	—	2.105 (0.766–5.784)	0.149
Disease duration						
Per 1-month increase	0.000 (−0.000 to 0.000)	0.726	—	—	1.004 (0.998–1.010)	0.161

Model K used ordinary least squares regression with K_score as a continuous outcome. Models A and P used multivariable logistic regression for positive attitude (A_positive) and good practice (P_good), respectively. Effect estimates are reported as β coefficients (Model K) and odds ratios (Models A and P) with 95% confidence intervals. — indicates that the predictor was not retained in the final model or was not estimated for that outcome. Reference categories are shown after ‘vs’. *P* values are two-sided.

CI, confidence interval; CNY, Chinese yuan; OLS, ordinary least squares; OR, odds ratio.

**Figure 2:**
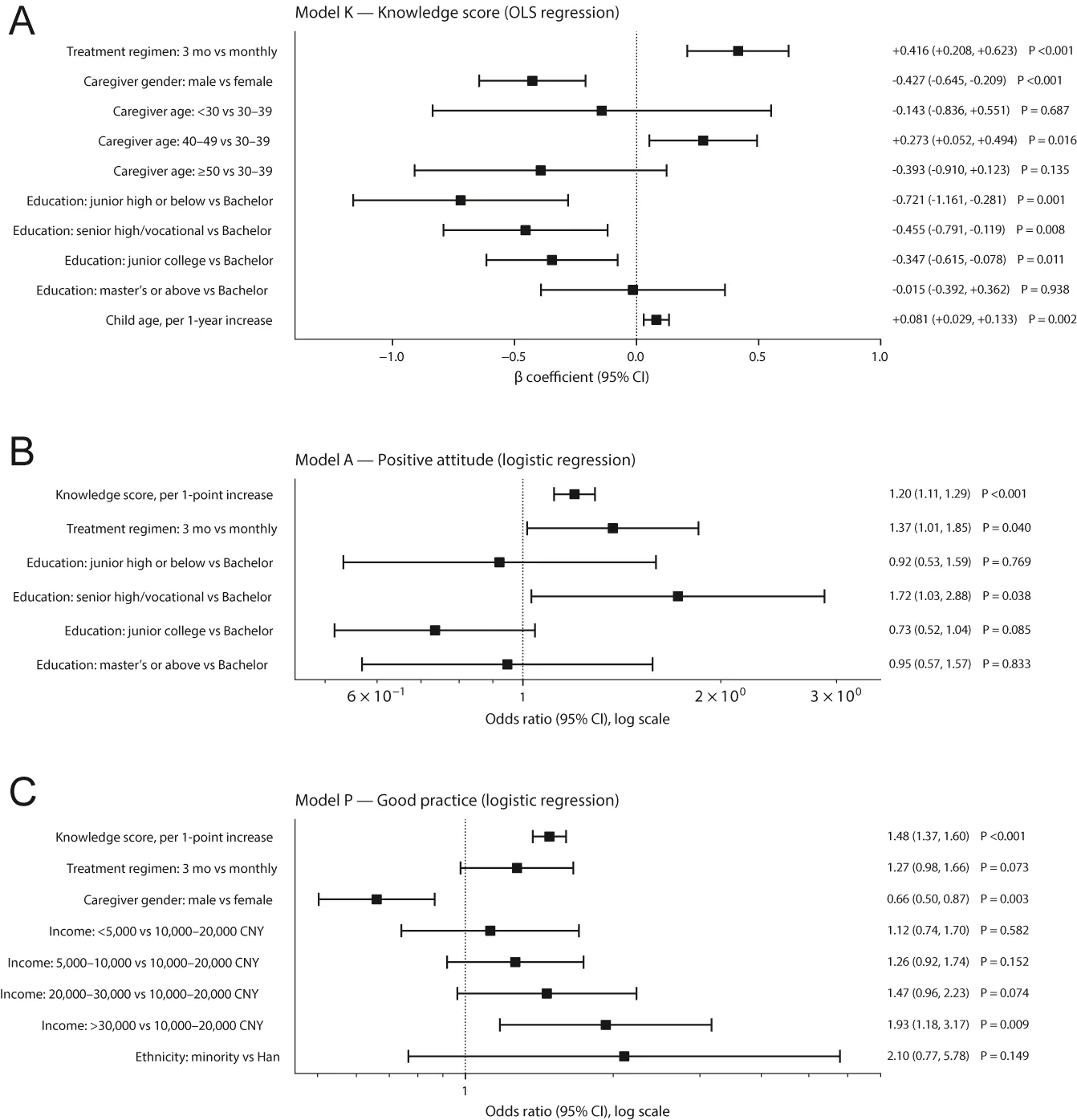
Forest plot of key determinants from multivariable regression models. Forest plot summarizing the associations of key determinants with KAP outcomes from three multivariable models: (A) ordinary least squares regression for knowledge score (K_score); (B) logistic regression for positive attitude (A_positive); and (C) good practice (P_good). Points indicate effect estimates (β for the OLS model and ORs for logistic models), and horizontal lines represent 95% confidence intervals. The dashed vertical reference line denotes no association (*β* = 0 for OLS; OR = 1 for logistic models). Full model estimates are provided in Table 3.

In the multivariable logistic regression model for positive attitude, each one-point increase in the knowledge score was associated with a higher likelihood of exhibiting a positive attitude (OR = 1.198, 95% CI: 1.115–1.287; *P* < 0.001). Treatment regimen remained independently associated with positive attitude, with caregivers receiving injections every three months showing a greater likelihood of positive attitudes than those receiving monthly injections (OR = 1.370, 95% CI: 1.015–1.849; *P* = 0.040). In addition, educational attainment showed heterogeneous associations with attitude, with caregivers educated to the senior high or vocational level demonstrating a higher likelihood of positive attitudes compared with those holding a bachelor’ s degree (OR = 1.721, 95% CI: 1.030–2.875; *P* = 0.038).

In the multivariable logistic regression model for good practice, knowledge score remained a strong independent determinant, with each one-point increase associated with substantially higher odds of good practice (OR = 1.483, 95% CI: 1.372–1.603; *P* < 0.001). Compared with monthly injections, the three-month regimen was associated with a higher likelihood of good practice, although this association did not reach conventional statistical significance (OR = 1.274, 95% CI: 0.978–1.659; *P* = 0.073). Household income also played a role, with caregivers from households earning more than 30,000 CNY per month exhibiting higher odds of good practice compared with those earning 10,000–20,000 CNY (OR = 1.931, 95% CI: 1.176–3.170; *P* = 0.009). No significant associations were observed for disease duration or ethnicity in the fully adjusted model.

### Decomposition of path effects

The observed-variable path model is illustrated in Fig. 3, and the decomposition of effects is presented in Table 4. Knowledge score was positively associated with positive attitudes (standardized *β* = 0.112, *P* < 0.001), and positive attitudes were in turn positively associated with practice (standardized *β* = 0.333, *P* < 0.001). After accounting for the mediating effect of attitudes, knowledge remained significantly and directly associated with practice (standardized *β* = 0.280, *P* < 0.001). In addition, knowledge exerted a significant indirect effect on practice through positive attitudes (standardized *β* = 0.037, *P* < 0.001), indicating that approximately 12% of the total effect of knowledge on practice (standardized *β* = 0.317, *P* < 0.001) was mediated via the attitudinal pathway.

**Figure 3:**
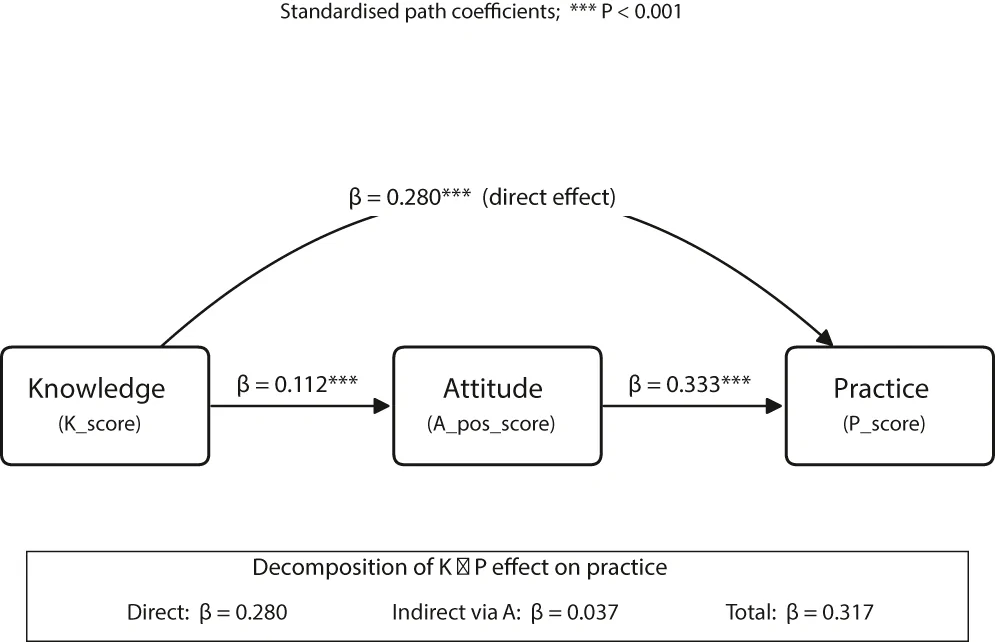
Path model depicting the KAP mechanism. ***P < 0.001; n.s., not significant.

**Table 4:** Decomposition of path effects among knowledge, attitude, and practice.

Effect	Standardized β	95% CI	*P* value
Knowledge → attitude	0.112	0.057–0.167	<0.001
Attitude → practice	0.333	0.288–0.378	<0.001
Knowledge → practice (direct effect)	0.280	0.229–0.335	<0.001
Knowledge → practice (indirect effect via attitude)	0.037	0.018–0.056	<0.001
Knowledge → practice (total effect)	0.317	0.264–0.375	<0.001

Knowledge, attitude, and practice correspond to K_score, A_pos_score, and P_score, respectively. Coefficients are standardized estimates from an observed-variable path model. Confidence intervals for the indirect and total effects were obtained by bootstrap resampling. The model was adjusted for caregiver and child covariates consistent with the multivariable models in Table 3.

CI, confidence interval.

## Discussion

### Main findings

Based on large-sample, internet-based survey data from eight centers, this study systematically transcends traditional descriptive analysis to provide a pioneering mechanistic roadmap of the ‘knowledge–attitude–practice’ chain among caregivers of children with CPP. We have achieved a significant breakthrough by identifying a synergistic link between treatment frequency and caregiver cognition. Notably, among treated caregivers, the every-3-month regimen was consistently associated with more favorable KAP profiles, including higher knowledge levels and a higher likelihood of positive attitudes compared with the monthly regimen (Supplementary Table S2; Table 3; Fig. 2). This pattern is clinically meaningful because less frequent dosing may reduce treatment-related burden and simplify follow-up logistics, facilitating superior information uptake and reinforcing caregivers’ confidence in a physician-led management framework.

Path analysis further validated that knowledge levels are not only positively correlated with positive attitudes and good practices but also exert an indirect effect on practice by enhancing positive attitudes (Tables 3 and 4, Figs 2 and 3). These findings redefine the logic of CPP management. Besides, they provide clearer treatment pathways that are essential to translate cognition into high-quality therapeutic outcomes. These findings align closely with the real-world clinical requirements of CPP management – specifically, the need for long-term follow-up, sustained adherence, and shared decision-making. Clearer treatment pathways and more comprehensible information are more likely to translate into stable caregiving behaviors and higher-quality therapeutic management (23).

### Comparison with previous research and interpretations

First, while overall knowledge and positive attitudes were relatively high, a distinct ‘gap’ remained in the practice dimension, particularly among families whose children were not yet receiving treatment. This ‘high awareness, low practice’ structure is common in KAP studies; knowledge and attitudes are necessary precursors, but behaviors are often constrained by resources, emotional burden, and accessibility (24). Second, ‘current treatment status’ emerged as a pivotal determinant across K, A, and P dimensions. This can be explained by the CPP management model: screening, diagnosis, GnRHa selection, and follow-up involve structured medical processes and frequent patient–provider communication. Once entering the treatment pathway, families receive significantly more structured information and behavioral prompts, enhancing both knowledge acquisition and execution intensity (1, 23). Third, the impact of knowledge on practice is partially mediated by attitudes (Table 4). This ‘knowledge promotes attitude, attitude drives behavior’ mechanism is consistent with behavioral science frameworks and fits the pathway emphasized in pediatric SDM literature: as parental understanding of the disease improves, they develop actionable beliefs and confidence, leading to more proactive engagement in follow-up tracking and adherence management (25, 26). Furthermore, differences in KAP based on caregiver characteristics (e.g. gender, education, and relationship to the child) suggest that CPP caregiving behaviors are a manifestation of familial labor division and health literacy variances. Recent research indicates that in time-sensitive contexts like CPP, deficiencies in parental health literacy and communication skills can exacerbate decisional conflict and psychological stress, further hindering behavioral implementation (16, 27).

### Clinical and public health implications

From a practical standpoint, interventions should prioritize two ‘highly modifiable’ groups: i) families who have not yet started treatment or are in early stages (characterized by information gaps and unclear pathways); and ii) families with lower education levels or those where grandparents are the primary caregivers (characterized by higher cognitive and execution burdens). To address these gaps, we advocate for a physician-led, synergistic management model that integrates standardized health education and proactive follow-up into outpatient pathways. We recommend using standardized tools – such as growth tracking templates, medication reminders, and adverse reaction management cards. Furthermore, in the emerging landscape of digital health, physicians should play a central role in the clinical standardization and calibration of AI-driven tools to ensure their medical accuracy and safety. Digital interventions offer a feasible way to extend these supports (20). At a broader level, the rising demand for precocious puberty treatment and COVID-19-related lifestyle changes highlight the urgency of building scalable family education and adherence support systems (28, 29).

### Strengths and innovations

This study has three primary strengths. First, the multicenter, internet-based approach enabled efficient, large-scale data collection of real-world caregiver experiences across diverse geographic and socioeconomic backgrounds in China, enhancing the generalizability of the findings. Second, the KAP measurement was embedded within the PRO-CaregiverCPP, the global first CPP-specific instrument strictly developed according to COSMIN guidelines – ensuring unprecedented content validity and clinical relevance compared with ad hoc questionnaires. Third, by decomposing direct and indirect effects through path analysis, we provide the first systematic decoding of the cognition–behavioral execution chain in CPP. This will offer a clearer scientific logic for physician-led interventions, which fill the gap between ‘comprehensible information’ and ‘actionable behaviors’.

### Limitations and future research

This study has several limitations. First, the cross-sectional design precludes strict causal inference; future longitudinal or intervention studies should verify whether improving K/A directly leads to better practices and clinical outcomes. Consequently, our team has initiated a large-scale, multicenter prospective cohort study to establish these causal links and evaluate long-term physiological trajectories. Second, internet-based surveys carry risks of selection bias and low-quality responses, necessitating even more rigorous data validation strategies in the future (30, 31). Our upcoming research will integrate the PRO-CaregiverCPP into an AI-enhanced management platform, where physicians will lead the tool’s calibration for personalized care. Finally, as CPP is associated with psychosocial risks, future research should integrate familial psychological support and child psychological outcomes into a more comprehensive assessment framework (14, 32).

## Conclusion

This study shows a clear, stepwise relationship between knowledge, attitudes, and practices among caregivers of children with CPP. Treatment status and health literacy are major factors influencing behavior. Caregiver understanding is important. However, physicians are the key to successful treatment. They provide structured education, standardize medication use, and manage care over time. These efforts help connect caregiver knowledge with long-term treatment adherence.

New technologies – such as digital follow-up platforms and artificial intelligence (AI) – offer new possibilities for CPP care. In this changing environment, physicians remain essential. They define clinical pathways, ensure digital tools are medically sound, and adjust them to fit real-world needs. Future interventions should provide clear information and simple follow-up tools. A physician-led, technology-assisted approach is especially important for families with untreated children or low health literacy. This can improve adherence and support shared decision-making over the long term.

## Supplementary materials



## Declaration of interest

The authors declare that there is no conflict of interest that could be perceived as prejudicing the impartiality of the work reported.

## Funding

This work was supported by the Beijing Top-Doctors Charity Foundation (no grant number assigned).

## Author contribution statement

SD, WL, and XY contributed equally to this work as co-first authors. YS and XL contributed equally to this work as co-corresponding authors. SD, WL, XY, YS, and XL contributed to the study conception and design. JC, SC, WG, QW, GZ, PZ, XM, CL, YL, ZJ, YY, JJ, and YP contributed to multicenter coordination, participant recruitment, data collection, and data interpretation. SD, WL, XY, YS, and XL drafted or critically revised the manuscript. All authors reviewed and approved the final manuscript and agree to be accountable for all aspects of the work.

## Data availability

The datasets generated or analyzed during the current study are not publicly available due to privacy and ethical restrictions related to real-world clinical data. De-identified participant-level data may be made available from the corresponding author upon reasonable request, subject to approval of a methodologically sound proposal and completion of an applicable data use agreement.

## Ethics approval and consent to participate

This study was conducted in accordance with the Declaration of Helsinki and relevant ethical standards. The study protocol was approved by the Ethics Committee of Tongji Hospital, Tongji Medical College, Huazhong University of Science and Technology (Approval No.: TJ-IRB202508001). Electronic informed consent was obtained from all participants before questionnaire completion.
