# Supplementary material for: An internet-based, multicenter, large-sample survey: knowledge, attitudes, and practices and pathway mechanisms among caregivers of children with central precocious puberty
Source: Endocr Connect. 2026 Oct 6;15(10):e260351. doi: 10.1530/EC-26-0351 (PMC13695397; doi:10.1530/EC-26-0351)
Supplement: Supplementary file 1 [file EC-26-0351_supplementary_materials.pdf]

Supplementary Material

Supplementary Table S1. Content and Response Options of the PRO-CaregiverCPP Questionnaire

Item No.	Item content	Response options
**Part I. Knowledge, Attitudes, and Practices (KAP)**		
1	Understanding of possible causes of central precocious puberty	I understand most cases have no identifiable primary cause / I do not understand well / I do not understand this question
2	Understanding of diagnostic methods for CPP	I understand diagnosis usually involves ultrasound, bone age, and hormone tests / I do not understand well / I do not understand this question
3	Understanding of the role of bone age in diagnosis and treatment	Yes, it helps assess future growth potential / No, I do not understand well / I do not understand this question
4	Perceived primary goal of GnRHa (“suppression injection”) treatment	Delay puberty to improve adult height and reduce psychological distress / I do not understand well / I do not understand this question
5	Awareness of potential psychological impact of CPP beyond height	Aware / Not aware / I do not understand this question
6	Awareness of differences between monthly and three-monthly GnRHa injections	Aware / Not aware / I do not understand this question
7	Awareness of common mild side effects during GnRHa treatment	Aware / Not aware / I do not understand this question

8	Preferred response to mild local injection reactions	Observe and inform doctor at next visit / Not sure how to handle / I do not understand this question
9	Belief that GnRHa treatment is necessary for children with CPP	Strongly disagree / Disagree / Neutral / Agree / Strongly agree / I do not understand this question
10	Concern about unknown long-term risks of GnRHa treatment	Strongly disagree / Disagree / Neutral / Agree / Strongly agree / I do not understand this question
11	Belief that GnRHa treatment is important for improving final adult height	Strongly disagree / Disagree / Neutral / Agree / Strongly agree / I do not understand this question
12	Perceived stress related to managing treatment-related tasks	Strongly disagree / Disagree / Neutral / Agree / Strongly agree / I do not understand this question
13	Monitoring and recording child's growth and pubertal development	Never / Rarely / Sometimes / Often / Always / I do not understand this question
14	Actively seeking information about CPP	Never / Rarely / Sometimes / Often / Always / I do not understand this question
15	Discussing illness and injections with the child in an understandable way	Never / Rarely / Sometimes / Often / Always / I do not understand this question
16	Adherence to physician-recommended treatment schedule	Never / Rarely / Sometimes / Often / Always / I do not understand this question
17	Difficulty helping the child cope with injection-related fear	Never / Rarely / Sometimes / Often / Always / I do not understand this question
	**Part II. Quality of Life Impact (QoL)**	

18	Feeling depressed or anxious due to child’s condition	Never / Rarely / Sometimes / Often / Always / I do not understand this question
19	Talking in depth with others about the child’s condition	Never / Rarely / Sometimes / Often / Always / I do not understand this question
20	Perceived impact of CPP on family life	Never / Rarely / Sometimes / Often / Always / I do not understand this question
21	Worry about unsatisfactory final adult height	Never / Rarely / Sometimes / Often / Always / I do not understand this question
22	Worry about unknown long-term side effects	Never / Rarely / Sometimes / Often / Always / I do not understand this question
23	Worry about bullying or social isolation at school	Never / Rarely / Sometimes / Often / Always / I do not understand this question
24	Worry about psychological unpreparedness due to early development	Never / Rarely / Sometimes / Often / Always / I do not understand this question
**Part III. Economic Burden (EB)**		
25	Financial burden of medical examinations	Strongly disagree / Disagree / Neutral / Agree / Strongly agree / I do not understand this question
26	Financial burden from travel and accommodation for hospital visits	Strongly disagree / Disagree / Neutral / Agree / Strongly agree / I do not understand this question
27	Overall treatment cost exceeding family’s financial capacity	Strongly disagree / Disagree / Neutral / Agree / Strongly agree / I do not understand this question
**Part IV. Treatment Preferences (TP)**		

28	Effectiveness in controlling pubertal development	Not important at all / Slightly important / Moderately important / Important / Very important / I do not understand this question
29	Maximizing improvement in future adult height	Not important at all / Slightly important / Moderately important / Important / Very important / I do not understand this question
30	Risk of treatment-related weight gain	Not important at all / Slightly important / Moderately important / Important / Very important / I do not understand this question
31	Type and likelihood of treatment side effects	Not important at all / Slightly important / Moderately important / Important / Very important / I do not understand this question
32	Reducing frequency of hospital visits	Not important at all / Slightly important / Moderately important / Important / Very important / I do not understand this question
33	Minimal interference with school and daily activities	Not important at all / Slightly important / Moderately important / Important / Very important / I do not understand this question
34	Lower injection frequency (e.g., every 3 months vs monthly)	Not important at all / Slightly important / Moderately important / Important / Very important / I do not understand this question
35	Child's fear of different injection regimens	Not important at all / Slightly important / Moderately important / Important / Very important / I do not understand this question

36	Lower overall treatment cost	Not important at all / Slightly important / Moderately important / Important / Very important / I do not understand this question
37	Physician’s professional recommendation	Not important at all / Slightly important / Moderately important / Important / Very important / I do not understand this question
38	Consistency between injection frequency and follow-up visits	Not important at all / Slightly important / Moderately important / Important / Very important / I do not understand this question

Supplementary Table S2. Univariate descriptive summary of KAP outcomes by caregiver and child characteristics (N=1471)

Variable	Group	N (%)	K_score (mean ± SD)	A_positive (%)	P_good (%)
**Caregiver gender**	Female	999 (67.9)	6.44 ± 2.13	73.2	62.5
	Male	472 (32.1)	6.03 ± 2.47	71.2	51.1
**Caregiver age group (years)**	<30	51 (3.5)	6.29 ± 1.86	58.8	54.9
	30 – 39	861 (58.5)	6.21 ± 2.28	73.9	58.4
	40 – 49	487 (33.1)	6.68 ± 1.99	72.9	62.6
	⩾50	72 (4.9)	5.07 ± 3.06	63.9	40.3
**Education level**	Junior high or below	104 (7.1)	5.67 ± 2.37	67.3	51
	High school / technical secondary	175 (11.9)	6.02 ± 2.29	77.1	52
	Junior college	306 (20.8)	6.18 ± 2.25	69.3	55.6
	Bachelor (ref)	746 (50.7)	6.51 ± 2.18	73.9	61.7
	Master or above	140 (9.5)	6.38 ± 2.38	70.7	65
**Monthly household income (CNY)**	<5,000	202 (13.7)	5.87 ± 2.36	69.8	51
	5,000 – 10,000	501 (34.1)	6.25 ± 2.21	71.5	59.7
	10,000 – 20,000 (ref)	447 (30.4)	6.27 ± 2.32	70.7	54.8
	20,000 – 30,000	190	6.49 ±	77.9	64.2

		(12.9)	2.26		
	>30,000	131 (8.9)	7.06 ± 1.75	79.4	73.3
**Relationship to child**	Mother	915 (62.2)	6.49 ± 2.05	74.3	64.3
	Father	439 (29.8)	6.16 ± 2.40	71.5	52.6
	Grandparents	56 (3.8)	4.93 ± 3.03	62.5	33.9
	Other	61 (4.1)	5.85 ± 2.54	62.3	44.3
**Child gender**	Girl	1,065 (72.4)	6.43 ± 2.08	74.8	60
	Boy	406 (27.6)	5.99 ± 2.61	66.5	55.7
**Currently receiving treatment**	No	286 (19.4)	4.53 ± 2.88	47.9	37.8
	Yes	1,185 (80.6)	6.74 ± 1.83	78.5	63.9
**Treatment regimen (among treated)**	Monthly	710 (60.3)	6.60 ± 1.93	76.3	61
	Every 3 months	468 (39.7)	6.97 ± 1.59	82.1	68.4

Table note: Values are descriptive summaries. Knowledge score corresponds to K_score; Positive attitude corresponds to A_positive; Good practice corresponds to P_good. Percentages for A_positive and P_good represent the proportion meeting the predefined thresholds. CNY indicates Chinese yuan.

Supplementary Table S3. Final multivariable model specifications selected by stepwise AIC

Model	Outcome	Final model specification (stepwise AIC)
**Model K (OLS)**	Knowledge score (K_score)	K_score ~ Treatment regimen (Every 3 months vs Monthly) + Caregiver age group (<30, 40 – 49, ≥50 vs 30 – 39) + Education (Junior high or below, High school/technical secondary, Junior college, Master's or above vs Bachelor) + Caregiver gender (Male vs Female)
**Model A (Logistic)**	Positive attitude (A_positive)	A_positive ~ K_score + Treatment regimen (Every 3 months vs Monthly) + Education (High school/technical secondary vs Bachelor)
**Model P (Logistic)**	Good practice (P_good)	P_good ~ K_score + Treatment regimen (Every 3 months vs Monthly) + Caregiver relationship (Father, Grandparents, Other vs Mother) + Monthly household income (<5,000; 5,000 – 10,000; 20,000 – 30,000; >30,000 vs 10,000 – 20,000 CNY)

Note: Variable selection was guided by the Akaike Information Criterion (AIC) using bidirectional stepwise procedures. Model K was fitted using ordinary least squares regression, whereas Models A and P were fitted using binary logistic regression. Reference categories are indicated after "vs". CNY indicates Chinese yuan.

Supplementary Table S4. Correlations among KAP continuous scores

	**K_score**	**A_pos_score**	**A_bar_score**	**P_score**
K_score	1	0.186	-0.024	0.39
A_pos_score	0.186	1	0.455	0.408
A_bar_score	-0.024	0.455	1	0.169
P_score	0.39	0.408	0.169	1

Table note: Values are Pearson correlation coefficients between continuous scores. K_score indicates knowledge; A_pos_score indicates positive attitude; A_bar_score indicates barrier/concern attitude; P_score indicates practice score.

Supplementary Figures

Figure S1. Item-level correct response rates for knowledge questions

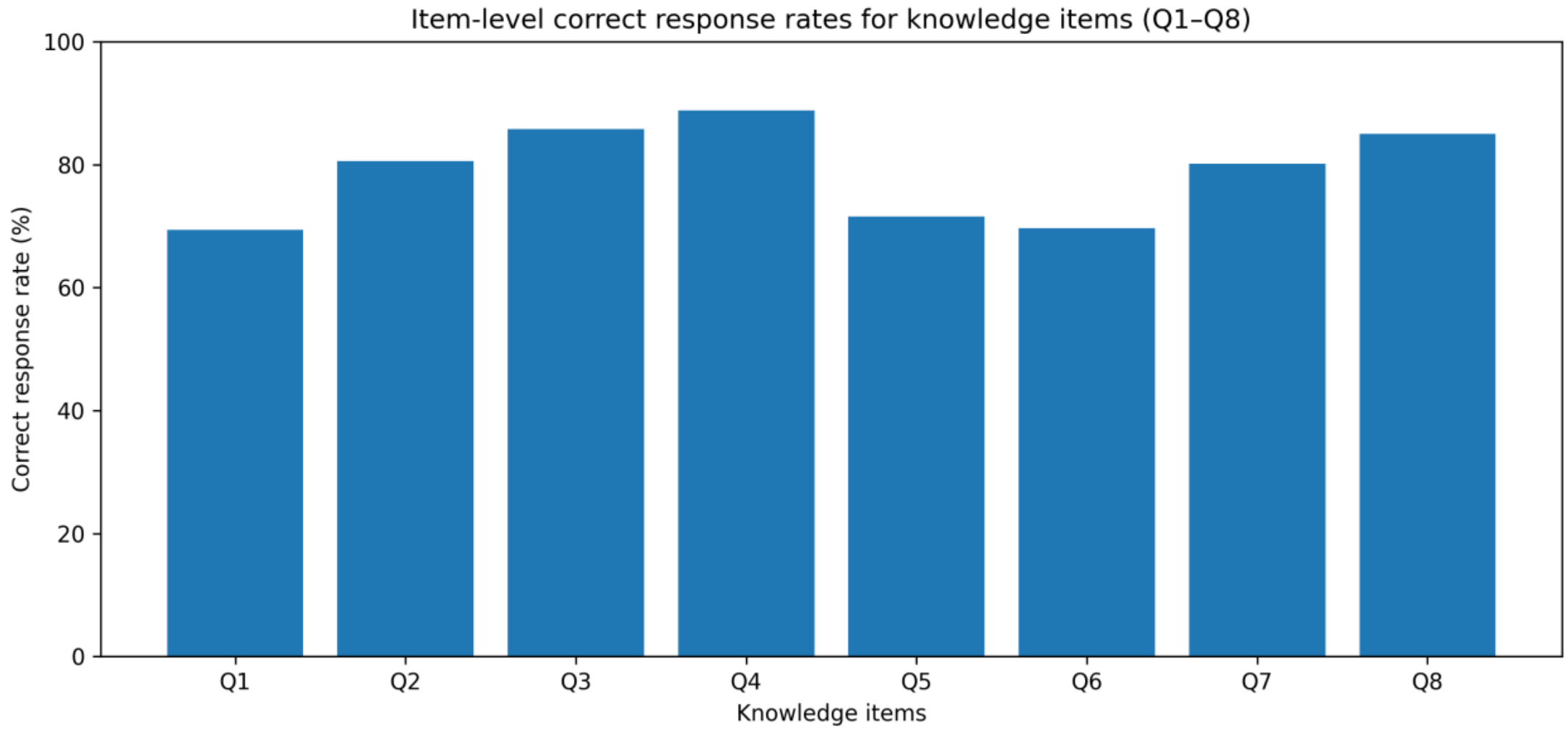

Figure legend: Bar chart showing the proportion of caregivers providing correct responses to each knowledge item (Q1–Q8). Correctness was scored dichotomously (correct/aware=1; incorrect/not sure=0), and item-level correct rates were calculated within the final analysis sample (N=1471). These item-level distributions complement the overall knowledge score reported in Table 2.

Figure S2. Distribution of responses across attitude items

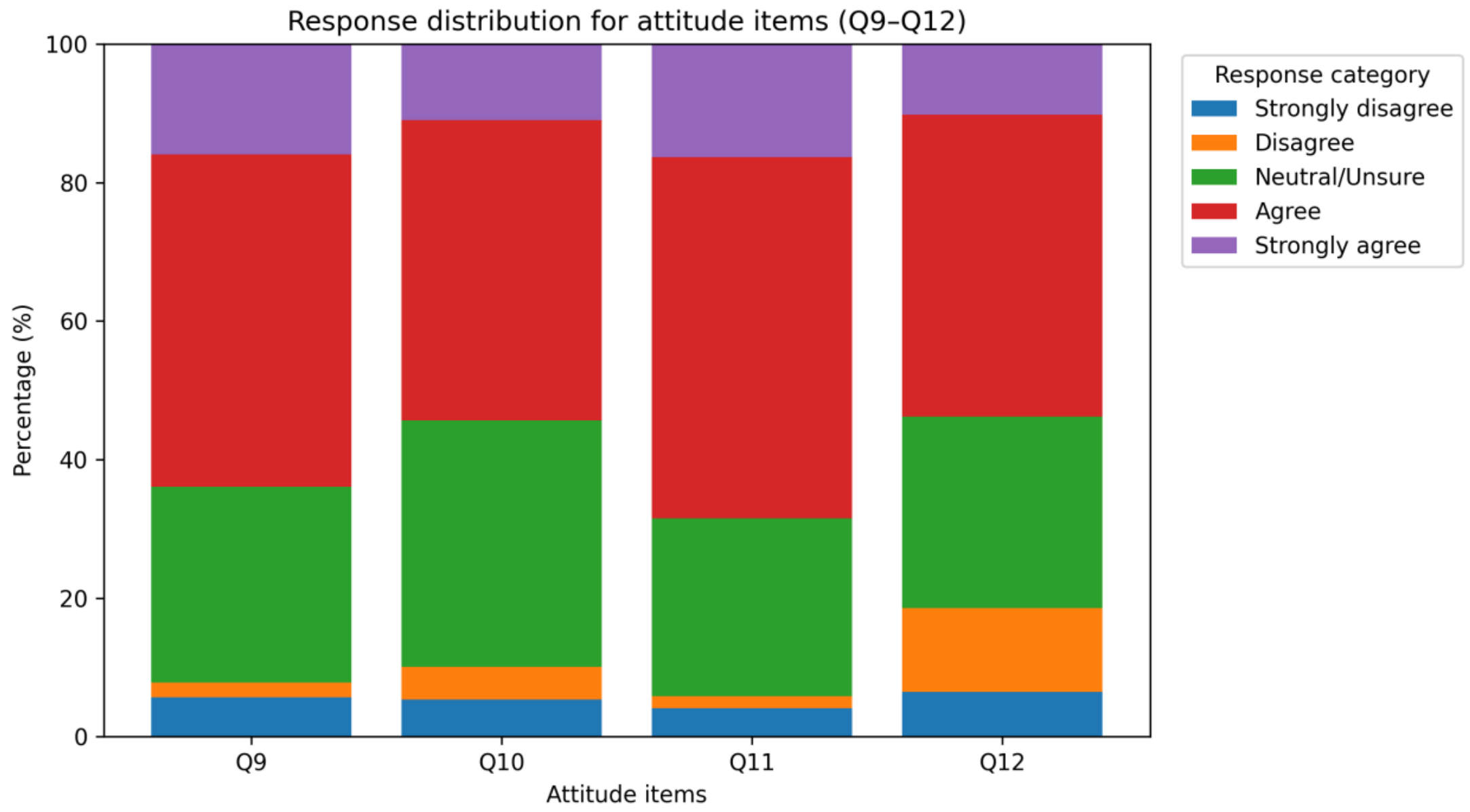

Figure legend: Stacked bar chart displaying the response distribution for attitude items (Q9–Q12) on a 5-point Likert scale. Percentages were calculated within the analysis sample (N=1471). The attitude construct was operationalized as two subdimensions—positive attitude (A_pos_score; Q9 and Q11) and barrier/concern attitude (A_bar_score; Q10 and Q12)—as summarized in Table 2.

Figure S3. Distribution of responses across practice items

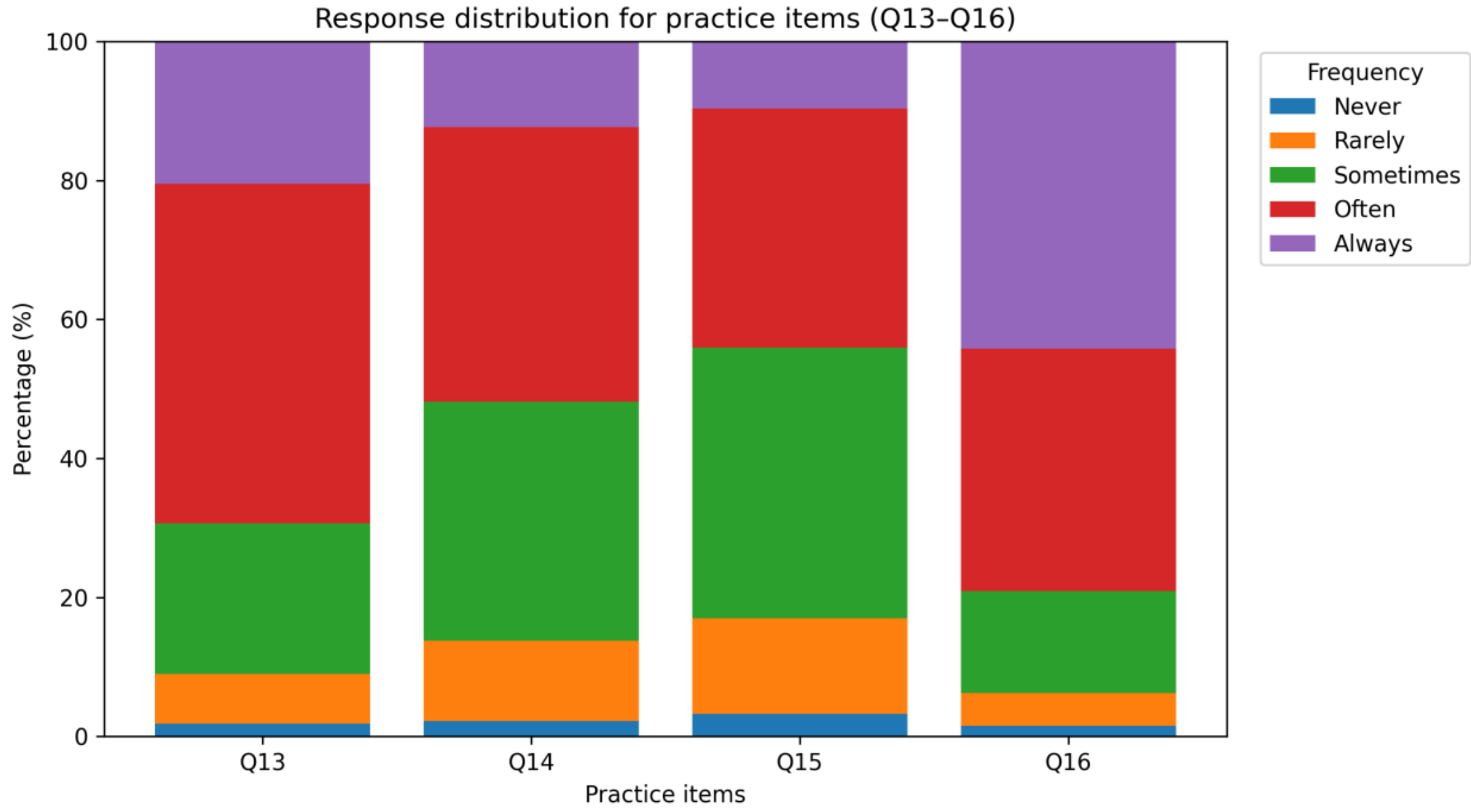

Figure legend: Stacked bar chart showing the frequency distribution for practice items (Q13–Q16) on a 5-point scale (from lower to higher frequency). Percentages were calculated in the final analysis sample (N=1471). The overall practice score (P_score) and the dichotomized outcome for good practice (P_good) are summarized in Tables 2 and 3.

Figure S4. Correlation heatmap among continuous KAP scores

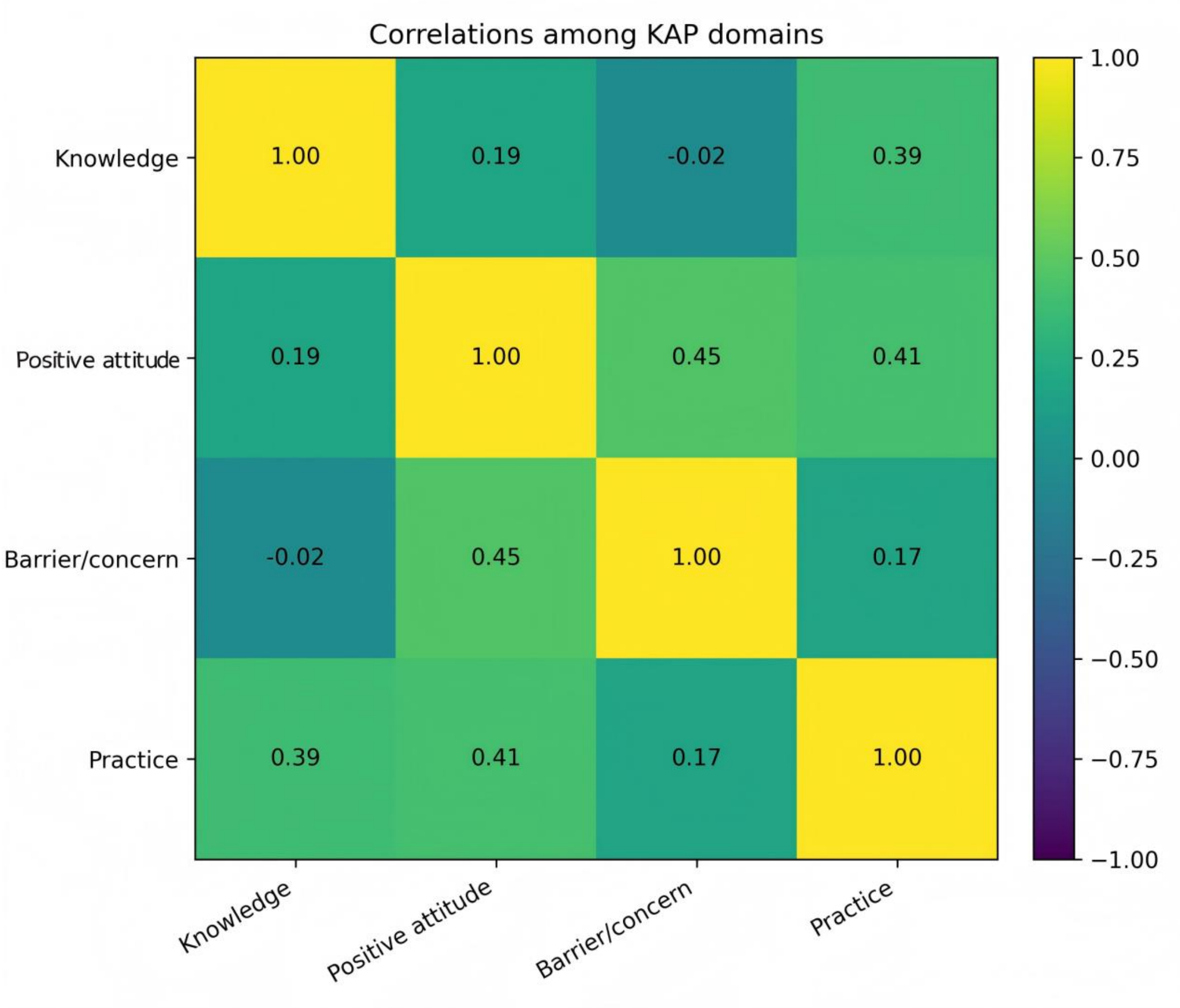

Figure legend: Heatmap of Pearson correlation coefficients among the continuous KAP scores: knowledge (K_score), positive attitude (A_pos_score), barrier/concern attitude (A_bar_score), and practice (P_score). Correlations were computed using the analysis sample (N=1471). Exact correlation coefficients are also provided in Supplementary Table S4.
